# Other Probable Explanations for Acute Neurological Deficits after the Removal of a Central Venous Catheter

**Published:** 2015-10-27

**Authors:** Ali Hosseinsabet

**Affiliations:** *Assistant Professor of Cardiology, Fellow of Echocardiography, Cardiology Department, Tehran University of Medical Sciences, Tehran Heart Center, Tehran, Iran. 1411713138. Tel: +98 21 88029731. Fax: +98 21 88029731. Email: ali_hosseinsabet@yahoo.com.*

Dear editor,

I read the article written by Ahmadi et al.^1^ published in the previous issue of your journal. In this invaluable article, the authors meticulously explained their observations on patients with acute neurological deficits after the removal of central venous catheters. However, there are some obscure points that should be pondered. 

Firstly, brain magnetic resonance imaging or repeated computed tomography scan for a better delineation of these neurological events was not done for most patients. Secondly, the patients should have undergone transesophageal study with contrast for the assessment of the possibility of the existence of the patent foramen ovale. Thirdly, the authors failed to consider the possibility of the entrance of the venous catheter into the left atrium.^2^^-^^4^ Central venous catheters may enter the left atrium accidentally in these patients, causing thrombosis. Also, the removal of catheters may result in embolization. Nonetheless, these two possibilities were ignored in this study. Another possibility discounted by the authors is the occurrence of paradoxical embolization through the pulmonary vasculature in the presence of a pulmonary arteriovenous fistula.^5^ The authors did not mention the time period during which these observations were collected, but it seems that these events were rare given the high number of surgical procedures performed in that center. Finally — in the explanation of their observations — the authors say, “Although only 4 patients in this study had evidence of intracardiac defects….”; however, elsewhere in the text the authors mention that bubble passage from the interatrial septum occurred only in 2 patients and that in 4 patients this study was not done or was negative. 

What should be taken into account in the explanation of these observations is the probability of pulmonary arteriovenous fistulae and accidental entrance of the central venous catheter into the left atrium. 
